# Advanced Interatrial Block Predicts Recurrence of Atrial Fibrillation and Ischemic Stroke in Elderly Patients With Hypertension

**DOI:** 10.3389/fphys.2022.913454

**Published:** 2022-06-16

**Authors:** Haijun Wang, Lili Cai, Yan Guo, Li Shuai, Yang Shi, Quanjin Si

**Affiliations:** ^1^ Department of Cardiology, The Second Medical Center and National Clinical Research Center for Geriatric Diseases, Chinese PLA General Hospital, Beijing, China; ^2^ Department of Laboratory Medicine, The Second Medical Center and National Clinical Research Center for Geriatric Diseases, Chinese PLA General Hospital, Beijing, China; ^3^ Department of the Third Health Care, The Second Medical Center and National Clinical Research Center for Geriatric Diseases, Chinese PLA General Hospital, Beijing, China

**Keywords:** atrial fibrillation, interatrial block, elderly, hypertension, stroke

## Abstract

**Background:** This study aimed to investigate whether advanced interatrial block (IAB) is a predictor of recurrent atrial fibrillation (AF) and/or ischemic stroke in elderly patients with AF and hypertension.

**Methods and objectives:** Five hundred and sixteen elderly inpatients (mean age 85.53 ± 9.08 years; 5.43% women) with concurrent paroxysmal AF and hypertension were enrolled in this retrospective observational study. Data on comorbidity, medication, digital electrocardiograms (ECG), and outcomes were obtained from the medical records and follow-up examinations. IAB was classified as partial IAB or advanced IAB according to 12-lead surface ECG analysis on admission. Advanced IAB was defined as a maximum P wave duration of >120 ms with biphasic (±) morphology in leads II, Ⅲ, and aVF by two blinded investigators. The endpoints were recurrent AF and ischemic stroke.

**Results:** We enrolled 120 patients (23.26%) with partial IAB and 187 (36.24%) with advanced IAB. The mean follow-up duration was 19 months. A total of 320 patients (62.02%) developed AF recurrence, and 31 (6.01%) experienced ischemic stroke. Significant predictors of advanced IAB in multivariate analysis were older age (>80 years), increased left atrial diameter (>40 mm), and being overweight (body mass index >25 kg/m^2^). In the multivariable comprehensive Cox regression analyses, partial IAB was associated with AF recurrence. Advanced IAB was an independent predictor of increased risk of AF recurrence and ischemic stroke.

**Conclusion:** Both partial and advanced IAB are associated with AF recurrence in elderly patients with hypertension. Furthermore, advanced IAB is an independent predictor of ischemic stroke.

## Introduction

Atrial fibrillation (AF) is one of the most common types of cardiac arrhythmias in elderly patients with hypertension and is associated with significant ischemic stroke and cardiovascular mortalities. Interatrial block (IAB) often occurs when a conduction delay occurs over Bachmann’s bundle or between the activation of the atrium. IAB was initially classified by Bayes de Luna and has recently often been defined according to IAB severity (Bayés de Luna et al., 2018; van der Does et al., 2021). Partial IAB is defined as a P-wave duration over 120 ms, and advanced IAB is defined as a P-wave morphology (positive/negative) in II, III, and aVF with a duration ≥120 ms. Advanced IAB has been reported as being associated with both new-onset and recurrent atrial fibrillation, left atrial electromechanical dysfunction, ischemic stroke, and cardiovascular mortality in some clinical scenarios (O'Neal et al., 2016a; Fujimoto et al., 2018; Rubio Campal et al., 2018; Lacalzada-Almeida et al., 2019). However, there is little data related to the prognosis of elderly patients with hypertension. The current study aimed to evaluate the role of advanced IAB in predicting recurrent AF and/or ischemic stroke in elderly patients with hypertension.

## Materials and Methods

### Patient Population

This was a retrospective observational study conducted at Chinese PLA general hospital in Beijing. All eligible patients were aged ≥65 years, of Chinese ethnicity, and diagnosed with hypertension and paroxysmal non-valvular atrial fibrillation between April 2014 and May 2016. Patients with the following conditions were excluded: 1) valvular AF such as post-mechanical valve replacement or moderate-to-severe rheumatic mitral valve stenosis; 2) severe renal insufficiency (estimated glomerular filtration rate of <30 ml/min/1.73 m^2^); 3) severe obstructive sleep apnea syndrome (apnea-hypopnea index of >10); 4) hyperthyroidism; 5) radiofrequency catheter ablation procedures for all types of atrial arrhythmia; 6) left atrial appendage occlusion procedures; and 7) cardiac pacemaker implantation. All participants provided written informed consent, and the study was approved by the institutional ethics committee.

### Electrocardiograms Analysis

**FIGURE 1:**
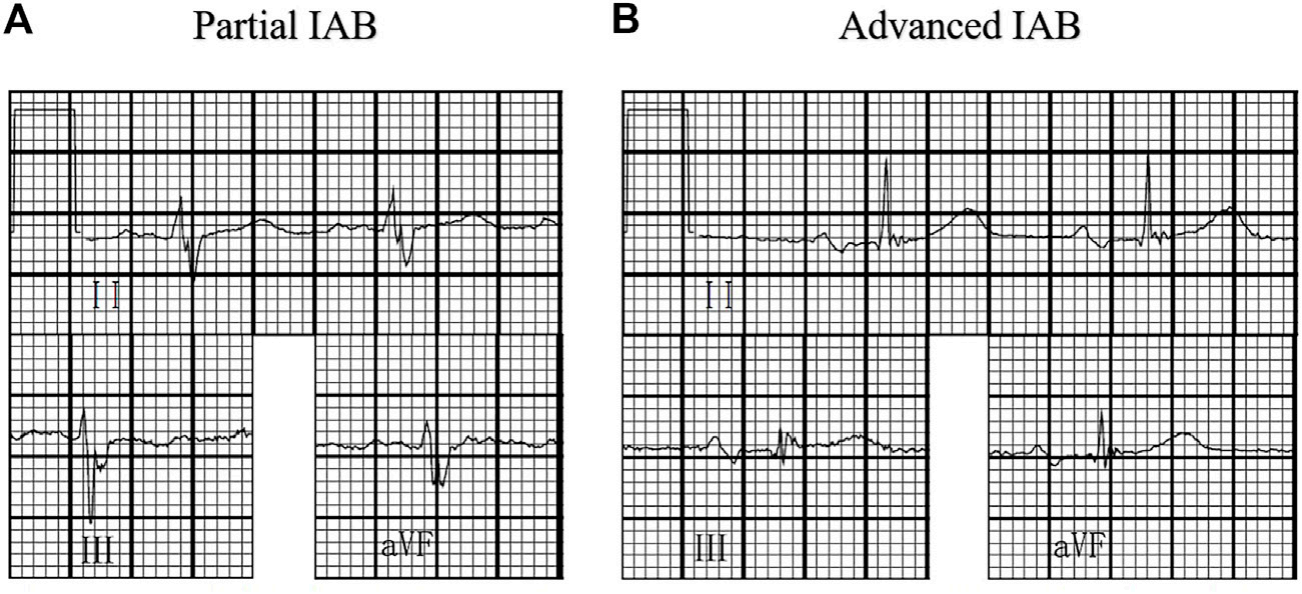
Interatrial block (IAB) degree. **(A)** partial IAB:P wave ≥120 ms without bimodal morphology;**(B)** advanced IAB:P wave ≥120 ms with bimodal morphology in inferior leads.

A surface standard 12-lead ECG (filter 150 Hz, 25 mm/s, 10 mm/mV) was recorded for each study patient at baseline and subsequent follow-up examinations and measured with digital calipers using the Muse Cardiology Information System. We amplified the ECG images up to 10 times their original size to define the P-wave duration and PR interval. IAB was classified according to the baseline 12-lead ECG by two independent blinded investigators as follows: 1) partial IAB was defined as P-wave duration longer than 120 ms without biphasic morphology in leads II, III, and aVF; and 2) advanced IAB was defined as P-wave duration longer than 120 ms and presented biphasic morphology in leads II, III, and aVF (Bayés de Luna et al., 2018) (Figure 1).

### Baseline Characteristics and Echocardiographic Analysis

All patients underwent two-dimensional transthoracic echocardiographic evaluation on admission. Echocardiographic parameters, such as ventricular ejection fraction (LVEF) and left atrial volume (LAV), were derived from the echocardiographic data. LVEF and LAV were measured using Simpson’s method and the area-length method, respectively. The left atrial volume index (LAVI) was calculated as the left atrial volume divided by the body surface area of each patient.

### Follow-Up and Outcomes

Baseline demographic characteristics, relevant medical histories, therapeutic procedures, cardiovascular events, and laboratory and imaging data were extracted from hospital electronic medical records. All patients were followed up at regular intervals or during all-cause hospitalization, with additional visits as required in the case of decompensation. The regular schedule of visits included a minimum of quarterly visits by the cardiovascular specialists. A 12-lead ECG was recorded at every visit or at any time when the patient had palpitations. A 24 h ECG monitor (Holter) was evaluated at least twice in patients with no recurrence, as necessary. Information on the endpoints was collected from the medical records and questionnaires answered by the patients. The primary endpoint was recurrent AF and was defined using ECGs or 24 h Holter electrocardiogram results. The secondary endpoint was AF-related ischemic stroke, which was defined as a sudden onset of neurological deficits or other relative symptoms lasting over 24 h and was associated with AF and confirmed using computed tomography or magnetic resonance imaging.

### Statistical Analysis

All statistical data were evaluated using IBM SPSS Statistics for Windows version 23.0 (IBM, NY, United States). Continuous variables are represented as mean ± standard deviation. Univariate analysis was performed using analysis of variance for continuous variables and the chi-square test for categorical variables. Kaplan–Meier estimates were used to compute the cumulative incidence of recurrent AF. Follow-up time was defined as the time between the baseline visit and recurrence of AF, time to loss to follow-up, death, or the end of the study period. We conducted multivariate logistic regression analyses to screen the independent risk factors for IAB. The Cox proportional hazard model was used to calculate the adjusted hazard ratio (HR) of risk factors for recurrent AF and ischemic stroke. Adjusted HRs with 95% confidence intervals (CIs) are reported separately. Statistical significance was set at *p* < 0.05.

## Results

### Baseline Characteristics of Study Patients

A total of 516 patients with paroxysmal AF and hypertension (women, 5.43%; mean age at baseline, 85.53 ± 9.08 years) were included in the study. During a mean follow-up of 19 months, 320 patients (62.02%) experienced AF recurrence, and 31 (6.01%) experienced ischemic stroke. The baseline characteristics of the patients, according to their IAB degree, are listed in Table 1. In this cohort, 209 (40.50%) patients had normal atrial conduction, 120 had partial IAB (23.26%), and 187 had advanced IAB (36.24%). In general, the more advanced the IAB, the higher the burden of the comorbidity. Patients with advanced IAB were significantly older compared to those with normal atrial conduction (87.95 ± 7.15 vs. 83.39 ± 10.12; *p* < 0.01). Participants with advanced IAB were more likely to have coronary artery disease, heart failure, or prior ischemic stroke than those with normal atrial conduction. They were also more likely to take diuretics and had higher values of PR intervals, P-wave duration, body mass index (BMI), left atrial diameter, and right atrial diameter. Participants with partial IAB were more likely to have a higher PR interval and P-wave duration than those with normal atrial conduction. Similarly, participants with advanced IAB were more likely to have anemia, P-wave prolongation, a larger left atrial diameter, a larger right atrial diameter, and a higher BMI than those with partial IAB. There was no significant difference in the use of antiarrhythmic drugs (including amiodarone and propafenone) and anticoagulants (recommended dose of rivaroxaban or dabigatran) among the three groups (partial/advanced and no IAB groups) and during follow-up.

**TABLE 1:** Baseline characteristics of the study participants with AF and hypertension according to the interatrial conduction.

Variables	No IAB (n = 209)	Partial IAB (n = 120)	Advanced IAB (n = 187)	*p-*value	*P*-value^a^	*p*-value^b^	*p*-value^c^
Age, (y)	83.39 ± 10.12	85.48 ± 8.96	87.95 ± 7.15	<0.001	0.121	<0.001	0.054
Gender, (females), n (%)	12 (5.74%)	7 (5.83%)	9 (4.81%)	0.897	0.973	0.681	0.695
Hypertension, n (%)	153 (73.21%)	97 (80.83%)	144 (77.01%)	0.283	0.119	0.383	0.426
Diabetes mellitus, n (%)	79 (37.80%)	48 (40.00%)	86 (45.99%)	0.242	0.693	0.099	0.302
Coronary artery disease, n (%)	131 (62.68%)	80 (66.67%)	141 (75.40%)	0.023	0.468	0.006	0.096
Heart failure, n (%)	18 (8.61%)	13 (10.83%)	33 (17.65%)	0.021	0.507	0.007	0.103
COPD, n (%)	60 (28.71%)	47 (39.17%)	61 (32.62%)	0.150	0.051	0.399	0.241
Stroke and TIA, n (%)	50 (23.92%)	36 (30.00%)	65 (34.76%)	0.060	0.227	0.018	0.386
Chronic kidney disease n (%)	29 (13.88%)	26 (21.67%)	37 (19.79%)	0.141	0.068	0.115	0.691
Anemia, n (%)	51 (24.40%)	20 (16.67%)	52 (27.81%)	0.080	0.101	0.441	0.025
ACEI/ARB, n (%)	85 (40.67%)	52 (43.33%)	67 (35.83%)	0.385	0.637	0.323	0.188
Beta-blocker, n (%)	102 (48.80%)	59 (49.17%)	107 (57.22%)	0.194	0.949	0.094	0.167
Anti-arrhythmic drugs, n (%)	63 (30.14%)	39 (32.50%)	48 (25.67%)	0.396	0.656	0.322	0.195
Diuretics, n (%)	56 (26.79%)	40 (33.33%)	87 (46.52%)	<0.001	0.209	<0.001	0.022
Anticoagulant drugs, n (%)	175 (83.73%)	108 (90.00%)	163 (87.17%)	0.261	0.115	0.335	0.451
PR interval, (ms)	181.02 ± 40.04	195.91 ± 43.17	204.10 ± 38.31	<0.001	0.004	<0.001	0.246
P-wave duration, (ms)	111.58 ± 4.33	123.61 ± 4.24	126.58 ± 6.42	<0.001	<0.001	<0.001	<0.001
BMI, (kg/m^2^)	23.66 ± 3.23	23.61 ± 3.56	24.69 ± 3.04	0.159	1.000	0.005	0.013
SBP, (mmHg)	130.89 ± 16.09	134.53 ± 18.21	131.51 ± 16.36	0.275	0.173	1.000	0.371
DBP, (mmHg)	68.27 ± 11.07	70.47 ± 12.15	69.96 ± 11.29	0.170	0.279	0.426	1.000
Left atrial diameter, (mm)	37.20 ± 3.97	37.67 ± 5.27	41.71 ± 6.20	<0.001	1.000	<0.001	<0.001
Right atrial diameter, (mm)	37.62 ± 2.96	38.29 ± 4.22	40.29 ± 5.19	<0.001	0.478	<0.001	<0.001
Serum creatinine, (μmol/L)	89.82 ± 51.61	98.76 ± 51.07	103.20 ± 76.94	0.093	0.624	0.097	1.000
NT-proBNP>450 pg/ml, n (%)	126 (60.29%)	74 (61.67%)	103 (55.08%)	0.435	0.805	0.295	0.254

Data are shown as the mean ± SD, or n (%). Abbreviation: AF, atrial fibrillation; COPD, chronic obstructive pulmonary disease; TIA, transient ischemic attack; ACEI, angiotensin-converting Enzyme Inhibitors; ARB, angiotensin-receptor blockers; BMI, body mass index; SBP, systolic blood pressure; DBP, diastolic blood pressure; NT-proBNP, N-terminal pro-brain natriuretic peptide.

a Normal atrial conduction vs. partial interatrial block.

b Normal atrial conduction vs. advanced interatrial block.

c Partial interatrial block vs. advanced interatrial block.

### Risk Factors for Advanced Interatrial Block

Table 2 shows the unadjusted and adjusted risk factors for advanced IAB. The strongest predictors of advanced IAB on admission in multivariate analysis were age of >80 years (odds ratio [OR] = 2.731, 95% CI: 1.629–4.577; *p* < 0.001), left atrial enlargement (left atrial diameter of >40 mm) (OR = 3.264; 95% CI: 2.206–4.829; *p* < 0.001), and BMI of >25 kg/m^2^ (OR = 1.700; 95% CI: 1.133–2.551; *p* = 0.010). Overall, this model explained 69.6% of the variability in the prevalence of advanced IAB.

**TABLE 2:** Results of univariate and multivariate logistic regression analysis for identifying predictors of advanced interatrial block.

Characteristics	Unadjusted OR (95%CI)	Univariate *p*-value	Adjusted OR (95%CI)	Multivariate *p*-value
Age>80 years	2.830 (1.747–4.583)	<0.001	2.853 (1.704–4.778)	<0.001
Left atrial diameter>40 mm	3.877 (2.655–5.660)	<0.001	3.392 (2.299–5.005)	<0.001
Body mass index >25 kg/m^2^	1.686 (1.168–2.433)	0.005	1.705 (1.137–2.555)	0.010
Coronary artery disease	1.714 (1.147–2.562)	0.009		
Heart failure	2.060 (1.216–3.491)	0.007		
Stroke/Transient ischemic attack	1.505 (1.021–2.221)	0.039		
Right atrial diameter>40 mm	2.341 (1.602–3.421)	<0.001		

### Incidence of Recurrent Atrial Fibrillation/Ischemic Stroke and Multivariate Cox Regression Model

The annual incidence of recurrent AF in patients with advanced IAB and hypertension was approximately 57.71 per 1,000 person-years, which declined stepwise to 35.02 per 1,000 person-years, and 15.92 per 1,000 person-years in patients with partial IAB and those with normal atrial conduction. Moreover, the annual incidence of ischemic stroke was highest in patients with advanced IAB (rate of 5.95 per 1,000 person-years), intermediate in patients with partial IAB (rate of 3.42 per 1,000 person-years), and lowest in patients with normal atrial conduction (rate of 1.31 per 1,000 person-years) (Table 3). There was no significant difference in the proportion of anticoagulant use between the stroke event and control groups (90.32% vs. 86.19%; *p* >0.05).

**TABLE 3:** Comparison of the rates of adverse events among the study groups.

Adverse Events	Normal Atrial Conduction		Partial interatrial block		Advanced interatrial block		*p* value
	No. of Events, n (%)	Incidence rate, (‰/yr)	No. of Events, n (%)	Incidence rate, (‰/yr)	No. of Events, n (%)	Incidence rate, (‰yr)	
Recurrent atrial fibrillation	73 (24.93)	15.92	82 (68.33)	35.02	165 (88.24)	57.71	<0.001
Ischemic stroke	6 (2.87)	1.31	8 (6.67)	3.42	17 (9.09)	5.95	0.032

**FIGURE 2:**
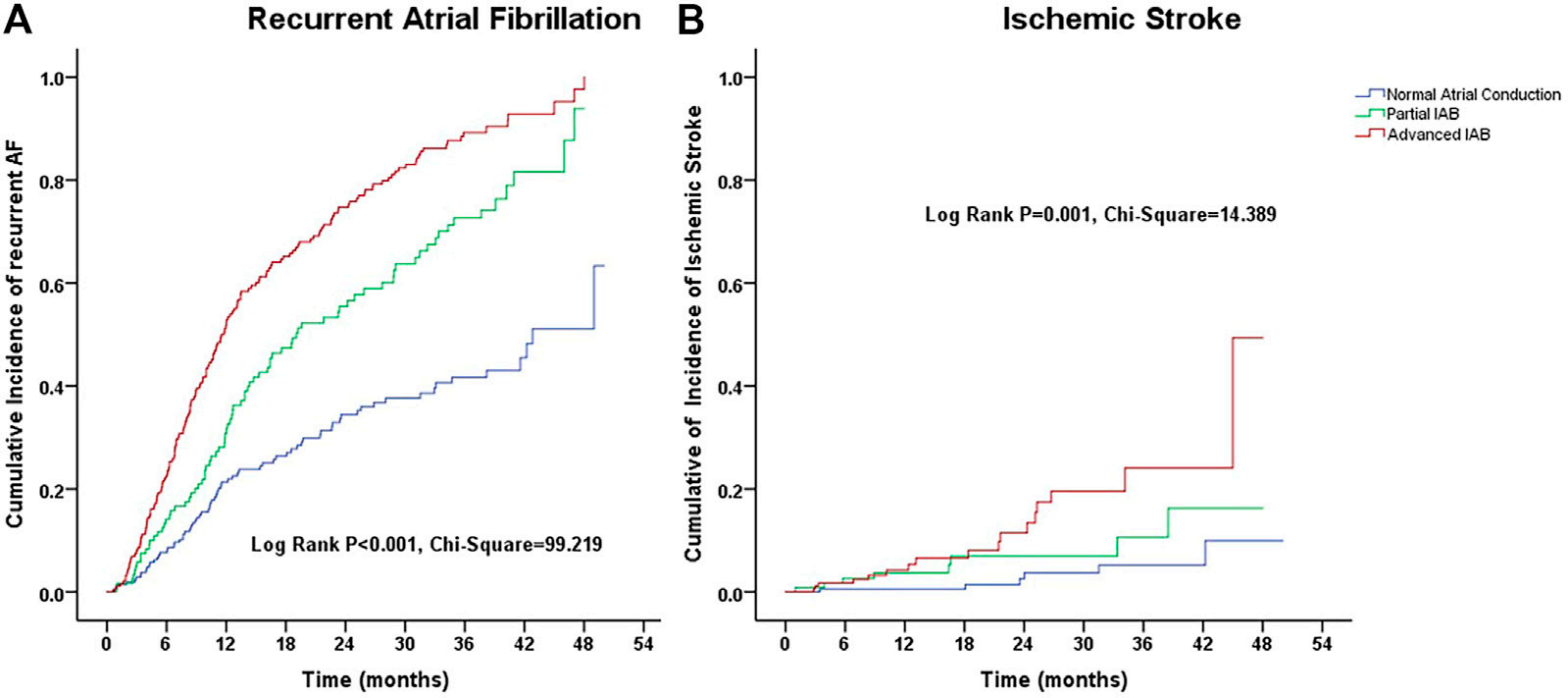
Kalpana-Meier estimate of cumulative incidences of **(A)** recurrent atrial fibrillation and **(B)** ischemic stroke according to the interatrial block (IAB) degree.

For univariate analysis, we observed significant differences in the Kaplan–Meier estimates of cumulative incidences of recurrent AF and ischemic stroke according to IAB severity (*p* < 0.01) (Figure 2).

A multivariate Cox regression model was applied, including IAB severity, age, sex, PR interval, BMI, hypertension, diabetes mellitus, coronary artery disease, heart failure, chronic obstructive pulmonary disease, prior stroke/transient ischemic attack, chronic kidney disease stage, anemia, left atrial diameter, right atrial diameter, N-terminal pro b-type natriuretic peptide (NT-proBNP) levels, and clinical medications. During the observation period, the crude HRs for recurrence of AF and ischemic stroke in the elderly AF patients with advanced IAB were higher than those with normal atrial conduction and remained significant (HR: 2.87, 95% CI: 2.12–3.88, *p* < 0.001; and HR: 4.57, 95% CI: 1.76–11.89, *p* = 0.002, respectively) after adjusting for potential confounders and demographics. Similarly, using multivariate analysis, we observed that partial IAB was also an independent risk factor associated with AF recurrence (HR: 2.165, 95% CI: 1.569–2.987; *p* < 0.001) but not for ischemic stroke (HR: 2.740; 95% CI: 0.944–7.958; *p* = 0.064) after adjusting for other risk factors. We observed no significant correlation between anticoagulant use and the risk of ischemic stroke (HR: 0.842; 95% CI: 0.255–2.773; *p* > 0.05). Additionally, age of ≥80 years, heart failure, left atrial diameter of >40 mm, right atrial diameter of >40 mm, anemia, and NT-proBNP levels of >450 pg/ml were all independent risk factors for AF recurrence (*p* < 0.05) (Table 4).

**TABLE 4:** Univariate and Multivariate Cox Regression Analyses of Risk Factors for Recurrent AF and Ischemic Stroke in elderly patients with hypertension during follow-up.

Adverse events	Univariate model			Multivariate model		
	HR	95% CI	*p* value	HR	95% CI	*p* value
Recurrence of AF						
IAB degree						
Normal atrial conduction	1.000 (reference)			1.000 (reference)		
Partial IAB	2.251	1.639–3.092	<0.001	2.165	1.569–2.987	<0.001
Advanced IAB	3.802	2.875–5.028	<0.001	2.871	2.123–3.881	<0.001
Age≥80 years	2.191	1.627–2.950	<0.001	1.396	1.013–1.926	0.042
PR interval≥200 ms	1.476	1.182–1.843	0.001			
Coronary artery disease	1.282	1.002–1.641	0.048			
Heart failure	2.385	1.780–3.195	<0.001	1.502	1.109–2.035	0.009
COPD	1.526	1.211–1.922	<0.001			
Stroke/TIA	1.498	1.187–1.889	0.001			
Chronic kidney disease	1.454	1.098–1.925	0.009			
ACEI/ARB	0.673	0.534–0.848	0.001	0.711	0.560–0.904	0.005
Left atrial diameter>40 mm	2.127	1.705–2.653	<0.001	1.337	1.022–1.748	0.034
Right atrial diameter>40 mm	1.950	1.561–2.435	<0.001	1.370	1.058–1.774	0.017
Anemia	1.556	1.210–2.001	0.001	1.574	1.210–2.049	0.001
NT-proBNP>450 pg/ml	1.335	1.064–1.674	0.012	1.375	1.089–1.736	0.008
Ischemic Stroke						
IAB degree						
Normal atrial conduction	1.000 (References)			1.000 (References)		
Partial IAB	2.855	0.987–8.261	0.053			
Advanced IAB	5.275	2.056–13.538	0.001	4.574	1.761–11.885	0.002
Age≥80 years	3.219	1.120–9.253	0.030			
Stroke/TIA	2.924	1.435–5.958	0.003	2.420	1.173–4.991	0.017
Diuretics	2.302	1.125–4.709	0.022			
Left atrial diameter>40 mm	2.096	1.031–4.262	0.041			

AF, atrial fibrillation; CI, confidence interval; IAB, interatrial block; COPD, chronic obstructive pulmonary disease; TIA, transient ischemic attack; ACEI, angiotensin-converting enzyme inhibitor; ARB, angiotensin II, receptor blocker.

Multivariate Cox regression model was applied including IAB severity, age, gender, PR interval, body mass index, hypertension, coronary heart disease, heart failure, stroke/transient ischemic attack, diabetes mellitus, chronic obstructive pulmonary disease, chronic kidney disease, anemia, left atrial diameter, right atrial diameter, NT-proBNP, and clinical medications.

## Discussion

In this single-center, respective observational study, the major findings were as follows: 1) the significant predictors of advanced IAB were age of >80 years, left atrial diameter of >40 mm, and BMI >25 kg/m^2^; 2) advanced IAB was significantly associated with an increased risk of recurrent AF and ischemic stroke in elderly patients with paroxysmal AF and hypertension; 3) partial IAB was also an independent predictor of recurrent AF but not of ischemic stroke.

IAB is rarely considered an independent, routine, and important electrocardiographic diagnosis despite its high prevalence and ease of diagnosis. Previous studies have found that the prevalence of IAB increased with age and could increase from 26% to 44% in patients with cardiovascular risk factors, rising to 50% in octogenarians (Martínez-Sellés, 2017; Escobar-Robledo et al., 2018; Skov et al., 2018). In the present study, the mean age of the participants was over 80 years. In our cohort, 187 patients (36.24%) had advanced IAB, and 120 (23.26%) had partial IAB. We further observed that IAB was often associated with increased P-wave duration and left atrial diameter in elderly patients. We believe that the high prevalence of advanced IAB in the present study could be due to the high prevalence of octogenarians and recognized stroke risk factors encompassed in the CHA_2_DS_2_-VASc (congestive heart failure, hypertension, age of ≥75 years, diabetes mellitus, stroke, vascular disease, age 65–74 years, and sex) risk score.

In the present study, we found that the significant predictors of advanced IAB were the age of >80 years, left atrial enlargement (left atrial diameter of >40 mm), and BMI of >25 kg/m^2^, which supports and extends the results of previous studies. A study by Bryce Alexander et al. demonstrated BMI of >30 kg/m^2^, male sex, and increased age (per 10 years increase) (OR = 1.46, 95% CI: 1.14–1.88) were significant predictors of IAB in patients with carotid and coronary artery disease (Alexander et al., 2018). The Atherosclerosis Risk in Communities (ARIC) study showed that age, male sex, BMI, and systolic blood pressure were all risk factors for advanced IAB development (O'Neal et al., 2016b). Another study revealed that an increased CHADS_2_ (congestive heart failure, hypertension, age >75 years, diabetes mellitus, prior stroke) score, coronary artery disease, and increased left atrial diameter were independently associated with IAB development. The association between IAB and degenerative diseases due to aging has been extensively studied. The mechanisms of the higher prevalence of IAB in obese patients than in those with normal weight may partly be due to an unhealthy lifestyle, diffuse ischemia, obstructive sleep apnea, and sympathetic nervous activation status (Baranchuk et al., 2017). Previous studies have confirmed the relationship between IAB and left atrial enlargement. In the present study, a left atrial diameter of >40 mm may be a more significant predictor of advanced IAB than other risk factors, as left atrial enlargement may be more directly related to atrial fibrosis and atrial conduction velocity, which are considered anatomical substrates of advanced IAB. Advanced IAB is probably a composite of impaired atrial conduction velocity and left atrial enlargement.

Several previous studies have found that IAB, especially advanced IAB, is strongly correlated with rapid atrial arrhythmias in different clinical scenarios and may predict ineffective cardioversion of atrial fibrillation (Relander et al., 2021). For example, Enriquez et al. reported that the overall recurrent AF in 61 patients without structural heart disease following pharmacological cardioversion was 36%, with a 90.9% recurrence in patients with advanced IAB (Enriquez et al., 2014). The ARIC study found that the incidence rate for advanced IAB was 2.27 per 1,000 person-years in 14,625 participants and confirmed a significant association between advanced IAB and an increased risk for AF (O'Neal et al., 2016). Recently, Wu et al. found the recurrence rate of paroxysmal AF after catheter ablation was higher in patients with advanced IAB than in those without advanced IAB (46.3% vs. 26.4%) (Wu et al., 2016). Furthermore, Skov et al. found that patients with advanced IAB and no cardiovascular disease (hypertension, heart failure, and ischemic heart disease) had a higher risk of AF than those with cardiovascular disease and no IAB (Skov et al., 2018). They also found that the addition of advanced IAB to a conventional risk model, including traditional cardiovascular risk factors, showed a high discriminative performance in the prediction of AF. In the present study, the incidence of recurrent AF in elderly patients with advanced IAB and hypertension was higher than that in those with normal atrial conduction (HR: 2.87, 95% CI: 2.12–3.88, *p* < 0.001), which supports and extends the results from previous studies. The mechanisms underlying the higher risk for recurrence of AF among patients with advanced IAB may partly be attributed to atrial fibrosis and atrial remodeling, which are considered anatomical substrates of advanced IAB, and play important roles in determining heterogeneous electrophysiological changes in atrial cardiomyocytes (Acampa et al., 2018; Bayés-de-Luna et al., 2020). IAB might result in electrical heterogeneity and impaired mechanical function of the left atrium and increased susceptibility to interatrial desynchrony and AF. Furthermore, cardiac ischemia, degenerative disease due to aging, infiltrative diseases, proinflammatory cytokines, the renin-angiotensin-aldosterone system, and oxidative stress may all disrupt the Bachmann’s bundle and interatrial desynchronization, thus promoting atrial cardiopathy and recurrence of AF (Kaireviciute et al., 2011; Martínez-Sellés, 2017; Escobar-Robledo et al., 2018). Some studies have further suggested that compromised left atrial function and heterogeneous electrophysiological changes might share a genetic underpinning with cardiomyopathy. The genetic risk of atrial fibrillation influences the left atrial structure prior to the diagnosis of AF (Ahlberg et al., 2021). The P-wave morphology distribution in atrial electrophysiology was also common in the early stage of arrhythmia and may be a cause rather than a consequence of AF (Holmqvist et al., 2011).

In this study, we observed a higher incidence of ischemic stroke in patients with advanced IAB than in those with normal atrial conduction. Advanced IAB, but not partial IAB, was an independent predictor of ischemic stroke after adjusting for potential confounders and demographics. Recent reports have shown that advanced IAB is associated with ischemic stroke. Evidence from prior studies has suggested that AF might serve as an intermediate event between advanced IAB and stroke. Subsequent rapid atrial arrhythmia may further lead to high risks of atrial cardiopathy, atrial dysfunction, and left atrial thrombosis (Lacalzada-Almeida et al., 2019; Istolahti et al., 2020). Patients with advanced IAB are usually more likely to have traditional risk factors for ischemic stroke and thromboembolism, such as hypertension, heart failure, diabetes, and aging. Some previous studies demonstrated that advanced IAB was associated with CHADS_2_ score, which was also a common independent predictor of IAB development and ischemic stroke (Wu et al., 2018). Furthermore, a recent study demonstrated that advanced IAB could predict all-cause mortality in ischemic stroke survivors with and without additional cardiovascular comorbidities (Baturova et al., 2019; Jacobsson et al., 2020; Prasitlumkum et al., 2020).

### Limitations

The current study has several limitations. First, the retrospective nature of this study and the relatively small sample size from a single center could have introduced selection bias. Second, recurrent AF was defined by ECGs and clinical examinations at every visit and 24-h Holter electrocardiograms in patients with no AF recurrence during follow-up. However, the recurrence rate of AF may have been underestimated, and more episodes of asymptomatic AF may be detected by longer rhythm monitoring. Third, IAB was classified according to the baseline 12-lead ECG, and changes in the performance of IAB during follow-up were not considered. Fourth, there is insufficient evidence to support our finding that advanced IAB could further improve the prediction efficiency of the CHA_2_DS_2_-VASc risk scoring system. Finally, we acknowledge that residual confounding remains a possibility, similar to that in other epidemiologic studies. Large-scale, multicenter, prospective studies with longer follow-up periods are required to validate our findings.

## Conclusion

Our results support the hypothesis that IAB is prevalent in elderly patients with hypertension. Both partial and advanced IAB were associated with AF recurrence in elderly patients with paroxysmal AF and hypertension. Furthermore, advanced IAB is an independent predictor of ischemic stroke.

## Data Availability

The data analyzed in this study is subject to the following licenses/restrictions: Confidentiality needs of patients’ clinical data. Requests to access these datasets should be directed to Haijun Wang, wanghj301@163.com.
